# Phosphoinositide 3-kinase inhibitor AS605240 ameliorates streptozotocin-induced Alzheimer’s disease like sporadic dementia in experimental rats

**DOI:** 10.17179/excli2019-1997

**Published:** 2020-01-06

**Authors:** Ramesh Alluri, Sivamallikarjuna Reddy Ambati, Kasiviswanth Routhu, Spandana Rajendra Kopalli, Sushruta Koppula

**Affiliations:** 1 Cognitive Science Research Initiative Lab, Dept. of Pharmacology, Vishnu Institute of Pharmaceutical Education and Research, Narsapur, Medak Dist., Telangana, 502313, India; 2 Aodh Lifesciences Pvt. Ltd., Tarnaka, Secunderabad-500017, Telangana, India; 3 Incozen Therapeutics Pvt. Ltd., Hyderabad-500078, Telangana, India; 4 Department of Bioscience and Biotechnology, Sejong University, Gwangjin-gu, Seoul 05006, Korea; 5 College of Biomedical and Health Science, Konkuk University, Chungju-Si, Chungbuk Do, 380-701, Republic of Korea

**Keywords:** Alzheimer's disease, AS605240, PI3K inhibitor, streptozotocin, intra-cerebroventricular injection, oxidative stress, cognition

## Abstract

The quest for chemical entities able to curb the action of the phosphoinositide 3-kinase, (PI3K)/protein kinase B (AKT) signaling pathways is evolving as a potential therapeutic strategy for the treatment and/or prevention of neurodegenerative disorders including Alzheimer's disease (AD). In this study, the effects of a PI3K inhibitor, AS605240 on cognitive dysfunction and antioxidative defense parameters against intra-cerebroventricular-streptozotocin (ICV-STZ)-induced rat model of sporadic AD was evaluated. ICV administration of a single dose of STZ (3 mg/kg) was performed to induce behavioral and biochemical changes in rats using the stereotaxic technique. Animals were administered with varying doses of AS605240 (5, 10 and 15 mg/kg) orally, 1 h before ICV-STZ on day 1 and continued once daily for four weeks. The behavioral parameters (passive avoidance and Morris water maze), antioxidative defense parameters, amyloid-beta (Aβ) protein expression by Western blotting and immunostaining technique were estimated in brain tissue. AS605240 dose-dependently and significantly (p < 0.05 and p < 0.01 and p < 0.001) improved ICV-STZ-induced cognitive impairment and attenuated the altered antioxidative related parameters including superoxide dismutase, lipid peroxidation, glutathione and nitrite levels. Further, the increased Aβ protein expression levels in brain tissue were markedly restored with AS605240 treatment. In conclusion, our study demonstrated that AS605240 exhibited immense potential in attenuating STZ-induced sporadic AD features in rats and may be developed as a therapeutic agent in the treatment and management of sporadic AD.

## Introduction

Alzheimer's disease (AD) is the principal cause of dementia in people over 65 years old, distressing millions of patients worldwide. Since its pervasiveness increases significantly with life expectancy the social impact of AD will be appalling if no effective therapy is developed in a near future (Brookmeyer et al., 1998[5]). The neuropathological hallmarks of AD include intracellular neurofibrillary tangles of hyper-phosphorylated tau, extracellular senile plaques and vascular deposits mainly composed of amyloid beta (Aβ) peptide, and selective synaptic and neuronal loss, particularly in the hippocampus and cerebral cortex (DeKosk, 2003[14]; Selkoe, 2001[49]; Masliah et al., 1994[32]). Accordingly, to the amyloid cascade hypothesis, abnormal deposition of Aβ is the central mechanism underlying pathological processes in AD (Hardy and Selkoe, 2002[21]). This notion is supported by the fact that all genetic mutations associated with familial AD increase the production of Aβ which is generated from the amyloid-β protein precursor (APP) through the cleavage by β- and γ-secretases (Tanzi and Bertram, 2005[57]; Zhang et al., 2010[61]). Currently existing classes of medication for the treatment of Alzheimer’s disease (AD), namely acetylcholinesterase inhibitors such as tacrine, rivastigmine, and galantamine and the N-methyl-D-aspartate receptor antagonist memantine have limited clinical benefits in terms of enhanced cognition and eventually do not prevent the disease progression (DeKosk, 2003[14]; Casey et al., 2010[7]; Stuchbury and Münch, 2005[54]). Therefore, exploring novel mechanistic strategies that prevent the pathological conditions of AD are quite necessary.

Mounting evidence suggests that the phosphoinositide 3-kinase, (PI3K)/protein kinase B (AKT) signaling pathway is directly impacted by Aβ exposure and is altered in AD brains. Aβ oligomers have been shown to modulate expression and density of insulin receptors that mediate growth and cell survival through the modulation of PI3K/Akt pathway (Zhao et al., 2008[62]). Further, several experiments on animal models revealed a marked increase in the phosphorylated Akt levels such as mammalian target of rapamycin (mTOR) and decreased cell-cycle inhibitors (p27kip1) in AD temporal cortex compared with control groups (Griffin et al., 2005[20]). These studies suggest that the components of insulin receptor and PI3K-Akt-mTOR pathway are affected in AD and correlate with altered cell cycle related events. Aβ oligomers alter the PI3K/Akt/mTOR pathway within neurons, and in turn promotes the expression of cell cycle proteins and the induction of neuronal cell cycle events which eventually leads to neuronal degeneration. In light of such reports, inhibition or regulation of PI3K/Akt signaling pathway might be a potential therapeutic strategy in ameliorating AD-like sporadic dementia.

AS605240 is a potent member of a new class of PI3Kγ-selective inhibitors with IC_50_ = 8 nM, and displaying over 30-fold more selectivity for PI3Kδ/β and 7.5-fold selectivity over PI3Ka in cell-free assays (Camps et al., 2005[6]; Cushing et al., 2012[13]). Pharmacologically, AS605240 is orally active and ATP-competitive (Peng et al., 2010[41]). Earlier studies showed that AS605240 attenuated the joint inflammation progression in lymphocyte-dependent and independent rheumatoid arthritis mouse models (Camps et al., 2005[6]). AS605240 also demonstrated a prominent role in regulating neuroblastoma tumors, autoimmune diabetes, autoimmune myocarditis, prevented bleomycin-induced pulmonary fibrosis, ameliorated concanavalin A-induced hepatic injury and dextran sodium sulfate-induced colitis in mice, prevented isoproterenol-induced hypertrophy and cardiac fibrosis, blocked the glomerulonephritis and increased the life span in various experimental animal models (Spitzenberg et al., 2010[53]; Azzi et al., 2012[1]; Barber et al., 2005[2]; Wang et al., 2009[58]; Jin et al., 2009[26]; Peng et al., 2010[41]; Song et al., 2011[51]; Wei et al., 2010[59]). However, the protective effects of AS605240 against neurotoxicity-induced experimental models of AD-like sporadic dementia was not studied.

Earlier reports indicated that a single intra-cerebroventricular (ICV) injection of streptozotocin (STZ; 3 mg/kg) is the most appropriate for induction of long term cognitive impairment, which can be used as a model of sporadic AD in experimental animals (Prickaerts et al., 1999[43]; Ramezani et al., 2016[45]). ICV-STZ experimental model exhibited neurochemical changes including behavioral and histological which are similar to human AD pathology (Dhull et al., 2012[15]; Ponce-Lopez et al., 2011[42]). Further, ICV-STZ model displayed neuroinflammation, reproduces amyloid pathologies as well as AD-like cognitive deficits (Correia et al., 2011[11]; Chen et al., 2013[9]; Nitsch and Hoyer, 1991[36]; Kraska et al., 2012[28]; Mehla et al., 2013[34]). 

In light of such reports, in the present study we aimed to investigate the effect of AS605240 on behavioral and cognitive impairments in ICV-STZ-induced rat model of sporadic AD. Further, the therapeutic benefits of AS605240 on antioxidative defense parameters and Aβ protein expression was also evaluated in rat brain tissues.

## Materials and Methods

### Chemicals

**Figure 1:**
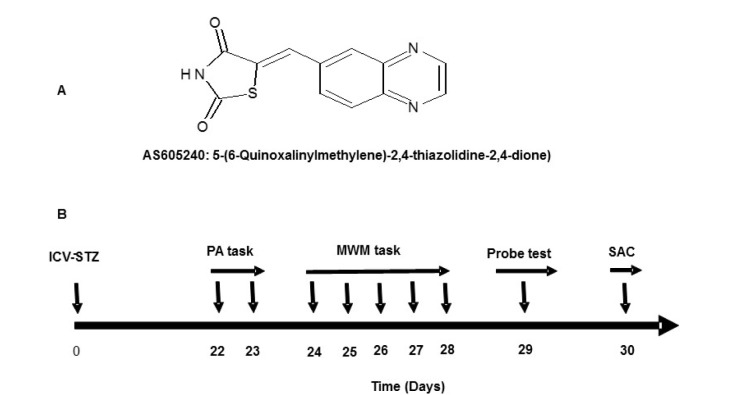
A: Structure and chemical name of AS605240. B: The experimental design, treatment schedule and intervals for estimation of various parameters. ICV-STZ=intra-cerebroventricular- streptozotocin, PA=Passive avoidance, MWM=Morris water maze, and SAC=sacrificed.

AS605240 (5-quinoxalin-6-ylmethylene-thiazolidine-2,4-dione (98 % pure, Figure 1A(Fig. 1)), glutathione (GSH), STZ, 5,5´-Dithiobis-2-nitrobenzoic acid (DTNB), 2-thiobarbituric acid (TBA), bovine serum albumin (BSA), and phosphate buffered saline (PBS), were purchased from Sigma (St. Louis, USA). Antibodies for Western blotting (WB) and immunohistochemistry were procured from Invitrogen, Camarillo, CA, USA. All the drug solutions were freshly prepared before administration.

### Animals

Wistar rats of either sex weighing 150-200 g (age, 8-12 weeks) were used in this study. Animals were housed in the Central Animal Facility of Vishnu Institute of Pharmaceutical Education and Research, under a 12 h light/dark cycle and specific pathogen free conditions with food and water *ad libitum*. Experiments were conducted according to the “Purpose of Control and Supervision of Experiments on Animals (CPCSEA) guidelines” and our Institutional Animal Care and Use Committee (No: 1358/ac/10/CPCSEA).

### Surgery and ICV-STZ administration

ICV injection of STZ was performed as previously described (Giacobini, 2006[18]). Briefly, rats were anesthetized with intraperitoneally (*i.p.*) injection of ketamine hydrochloride (70 mg/kg, *i.p.*) immediately followed by diazepam (4 mg/kg, *i.p.*). The animal was laid on surgery board, and the head was positioned straight, hair was trimmed, and a midline sagittal incision was made in the scalp. Two holes were drilled in the skull on both sides over the lateral ventricles using the following coordinates: 0.8 mm posterior to the bregma, 1.5 mm lateral to the sagittal suture, and 3.6 mm beneath the surface of the brain. Through a skull hole, a 28-gauge Hamilton syringe of 10 µl attached to a micro-injector unit and piston of the syringe was lowered manually into the lateral ventricle. The lesioned groups received bilateral ICV injection of STZ (3 mg/kg b.w) in artificial cerebrospinal fluid (ACSF) as described in earlier reports (Paxinos and Watson, 1986[40]). Similar surgical procedures were followed for control group with ACSF injections instead of STZ. During the operation and recovery, rats were held on a warm pad. For recovery, the rats were housed singly until their behavior returned to normal.

### Animal groups and drug treatments

After acclimatization for one week, animals were randomly separated into seven groups (n=12). The control Group I received a single ICV infusion of ACSF. Group II was infused with a single dose of STZ on day 1 (3 mg/kg, ICV). Group III, IV and V were infused with a single dose of STZ (3 mg/kg, ICV) on day 1 and immediately after STZ infusion were treated once a day orally (p.o.) with AS605240 (5, 15 and 25 mg/kg/day, respectively) for 21 days. Group VI was infused with a single dose of STZ (3 mg/kg, ICV) on day 1 and immediately after STZ infusion was treated once a day orally (p.o.) with a standard drug, donepezil (0.1 mg/kg; p.o.) for 21 days. AS605240 and donepezil were dissolved in vehicle (PBS) and the solution was adjusted to 10 ml/kg of body weight. Animals were subjected to the behavioral and biochemical tests after the fourth week of STZ or ACSF infusions.

### Behavioral studies

Behavioral tests started fourth week after ICV-STZ infusion and final administration of AS605240 or donepezil. The experiments were executed between 9:00 am and 4:00 pm in the laboratory at standard optimal conditions. All the tests were performed and analyzed by a researcher blind to the experiment.

### Passive avoidance test

On days 22 and 23, the rats were screened for memory retention deficits using a passive avoidance test (Raghavendra and Kulkarni, 2001[44]). The apparatus consisted of a two-compartment dark/light shuttle box with a behead door splitting the compartments. The dark compartment had a stainless steel shock grid floor. During the acquisition trial, each rat was positioned in the light chamber. After a 60 s habituation period, the guillotine door was unlocked, and the initial latency of the animals to get into the dark chamber was recorded. Rats with an initial latency time of more than 60 s were disqualified from further experiments. Immediately after the rat had got into the dark chamber, the guillotine door was locked and an electric foot shock (75 V; 0.2 mA, 50 Hz) was treated to the floor grid with a stimulator for 3 s. Five seconds later, the rat was taken from the dark chamber and returned to home cage. After 24 h, the retention latency time was measured in the same way as in the acquisition trial, but the foot shock was not given and the latency time was recorded to a maximum of 600 s. Short latencies indicate poorer retention.

### Morris water maze (MWM) test

After completion of passive avoidance test, spatial learning and memory of the animals were tested in a Morris water maze (Morris, 1984[35]). It contained of circular water tank (130 cm diameter, 62 cm height) filled 40 cm with water (25 ± 2 °C). A non-toxic paint was used to render the water cloudy. The pool was divided into four equal quadrants, labeled North, South, East, and West. An escape platform (10 cm in diameter) was hidden 2 cm underneath the surface of the water at a fixed location in one of the quadrants. The platform is placed in the same quadrant during the complete experiment. Before the training is initiated, rats were permitted to swim freely in the pool for 60 s with the platform. After climbing the platform, the animal remained there for 30 s before the instigation of the next trial. If the rat failed to detect and reach the escape platform within the maximum allowed time of 60 s, it was gently positioned on the platform and permitted to remain there for the same interval of time. The time for each platform (latency in seconds) was noted. Twenty-four hours after the acquisition phase, a probe test was led by removing the platform. Rats were permitted to swim freely in the pool for 60 s. The time spent in the target quadrant, which had previously contained the hidden platform, was noted. The time spent in the target quadrant indicates the degree of memory consolidation which had taken place after learning.

### Biochemical analysis

#### Tissue preparations

Twenty-four hours after the MWM tests, animals were euthanized under deep anesthesia, and their brains were extracted quickly and kept over a glass plate on ice. Hippocampus and cerebral cortex were dissected carefully as described previously (Paxinos and Watson, 1986[40]). Six brains were dissected to half along the midline, and frozen on dry ice immediately. One hemisphere was used for WB where in the tissues were homogenized in lysis buffer and the other hemisphere were separately homogenized in 10 mM tris-buffer (pH 7.4) containing protease inhibitors. The homogenate was centrifuged at 800 ×g for 5 min at 4 °C to separate the nuclear debris and used for estimation of lipid peroxidation (LPO). The supernatant was further centrifuged at 10,000 ×g for 20 min at 4 °C to get the post mitochondrial supernatant (PMS), which was used for other biochemical assays. The brains of other six rats in each group, after transcardial perfusion with saline followed by 4 % paraformaldehyde, were fixed in 3.7 % formaldehyde in PBS 1X solution. The brains were embedded in paraffin and cut into thin section of 5 µm each for immunohistochemistry analysis.

#### Measurement of LPO

The estimation of thiobarbituric acid-reactive species (TBARS), an index of LPO in the brain, was performed as reported previously (Ohkawa et al., 1979[38]). Briefly, 0.1 ml of supernatant was incubated with 0.5 ml Tris-HCl (0.1 M, pH 7.4) for 2 h. To this, 1 ml of trichloroacetic acid (10 %, w/v) was added and centrifuged at 1,000×g for 10 min. To 1 ml supernatant, 1 ml (0.67 %, w/v) thiobarbituric acid (TBA) was added and kept in the boiling water bath for 10 min, cooled, and added 1 ml distilled water. The amount of lipid peroxidation products was measured by reaction with TBA at 532 nM using the T60 UV/VIS spectrophotometer (PG Instruments Limited, India).

#### Effect on brain nitrite level

The brain nitrite levels in supernatant was determined using a colorimetric assay as described previously (Green et al., 1982[19]). Briefly, equal volumes of supernatant and Greiss reagent were mixed, the mixture was incubated for 10 min at room temperature in the dark, and the absorbance at 540 nm was determined with T60 UV/VIS spectrophotometer (PG Instruments Limited, India). The concentration of nitrite was expressed as micromoles per milligram protein.

#### Effect on brain GSH level

GSH content in the brain was estimated according to the method described by Ellman (1959[16]). Briefly, 1 ml supernatant was precipitated with 1 ml of 4 % sulfosalicylic acid at 4 °C for 1 h. The samples were centrifuged at 1,200×g for 15 min at 4 °C. To 1 ml of this supernatant, 2.7 ml of phosphate buffer (0.1 M, pH 8) and 0.2 ml of 5,5-dithio-bis (2-nitrobenzoic acid) were added. The absorbance of the yellow color developed was measured at 412 nm using T60 UV/VIS spectrophotometer (PG Instruments Limited, India).

#### Effect on brain superoxide dismutase (SOD) activity

SOD activity was determined according to the previous method (Beauchamp and Fridovich, 1971[4]). One unit (U) of SOD activity is defined as the amount of enzyme required to inhibit the rate of nitro blue tetrazolium (NBT) reduction by 50 %. The absorbance of the reaction mixture was measured at 550 nm using T60 UV/VIS spectrophotometer (PG Instruments Limited, India) and enzymatic activity was expressed as U/mg protein.

### Western blot analysis 

Western blot analysis was performed according to the manufacturer's protocols (Invitrogen, Carlsbad, CA). The protein concentrations of the homogenized brain sections were measured using the Bradford protein assay (Bio-Rad Laboratories, Hercules, CA, USA). Proteins were resolved by 4-20 % SDS-PAGE gel electrophoresis and electro transferred onto nitrocellulose membranes. After blocking with 5 % non-fat milk, the membranes were subjected to immunoblot analysis by incubation overnight with primary antibodies of rabbit polyclonal anti-APP (catalog number: 51-2700; 1:1000 dilution; Invitrogen, Camarillo, CA, USA) and anti-β-tubulin (Invitrogen, Camarillo, CA, USA, catalog number: 32-2600; dilution: dilution 1:4000) to normalize for the amount of protein loaded. Blots were washed with PBS and detected utilizing goat anti-rabbit or goat anti-mouse secondary antibody conjugated with horseradish peroxidase (dilution, 1: 100,000; A32735; Invitrogen, Camarillo, CA, USA). Protein bands were visualized using chemiluminescence reagent (ECL Plus) and exposed on X-ray film (Kodak, Rochester, NY, USA). The densitometric analysis of protein bands were imaged with alpha imager gel documentation system (FluorChem E; Cell Biosciences, USA) with β-tubulin as a loading control.

### Immunocytochemistry 

For immunohistochemistry, brain sections were deparaffinized, washed (3-10 min) with 0.1 M phosphate buffered saline (PBS) (pH 7.4) and incubated in blocking solution (PBS containing 0.3 % Triton X-100, 0.1 % BSA, and 2 % normal goat serum) for 2 h at room temperature. Primary and secondary antibodies were diluted in blocking solution. Following blocking, sections were incubated with polyclonal rabbit antibody to β-amyloid 42 (1:1000, Invitrogen) overnight at 4 °C with gentle shaking. After the overnight incubation, sections are rinsed with PBS twice, and the immunoreactivity was detected by incubating for 1 h in horseradish peroxidase-conjugated goat anti-rabbit IgG (Invitrogen, 1: 1,000). After incubation the sections were washed three times with PBS and 100 µl 3,3'-diaminobenzidine (DAB) was added as a color reagent solution to the sections, and the reaction was observed under a microscope. The sections were counterstained with 0.1 % Cresyl violet (Nissl stain). Sections were dehydrated with gradient alcohol and sealed with neutral gum. The appearance of brown DAB reaction products was identified as positive staining.

### Statistical analysis

Data are expressed as means ± standard error of the mean (S.E.M.). The behavioral and biochemical data were analyzed by one-way analysis of variance (ANOVA) followed by Bonferroni’s post hoc test using GraphPad Prism 5 software (GraphPad Software Inc., USA). In all experiments, a value of p < 0.05 was considered as statistically significant.

## Results

### Effect of AS605240 on memory retention deficit in passive avoidance test 

**Figure 2:**
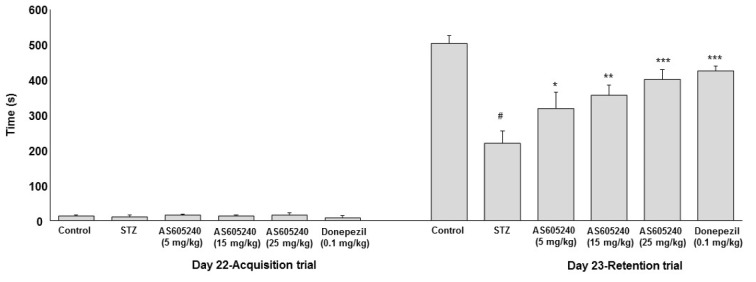
Effect of AS605240 on memory performance passive avoidance test in ICV-STZ rats. Latency time was recorded to a maximum of 600 s. Values are expressed as means ± S.E.M. (n=12). STZ-induced rats showed shorter retention latency compared with control group (^#^p < 0.001 vs control group). AS605240 (5, 15 and 25 mg/kg) and donepezil treatment in ICV-STZ-induced groups dose-dependently and significantly increased the retention latencies compared with STZ alone treated group (^*^p < 0.05, ^**^p < 0.01 and ^***^p < 0,001, respectively). STZ: Streptozotocin

To evaluate the effect of AS605240 on memory performance in passive avoidance task, the acquisition latency (AL) and retention latency (RL) in ICV-STZ rats was studied (Figure 2(Fig. 2)). On day 22, the AL values showed no significant differences across the groups tested. However, the RL in STZ-induced rat group on day 23 was significantly (p < 0.001) lower (220 ± 29 s) than those recorded for the control group (504 ± 33 s). The RL times in AS605240 treated groups improved significantly (p < 0.05 and p < 0.001) in a dose-dependent manner in antagonizing memory deficits induced by ICV-STZ (5 mg/kg, 320 ± 37; 15 mg/kg, 356 ± 27s; 25 mg/kg, 402 ± 25s). The standard donepezil treated group also showed significant (p < 0.001) effect compared with ICV-STZ alone treated group and the values are comparable with AS605240 treated at 25 mg/kg dose (p < 0.01, 421 ± 38s). 

### Effects of AS605240 on escape latency in rats by MWM test

**Figure 3:**
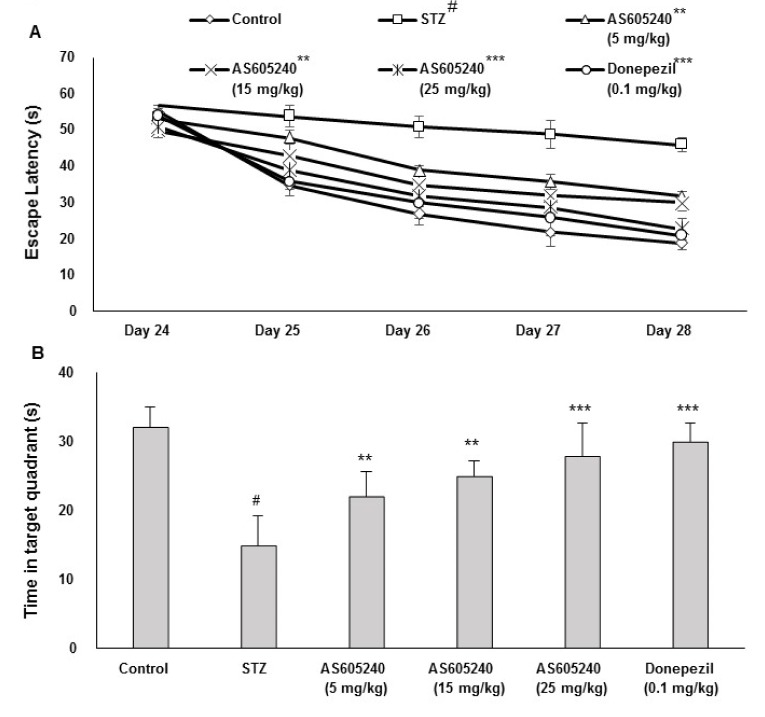
Effect of AS605240 on memory performance in Morris water maze (MWM) task in ICV-STZ-induced rats. A: Supplementation on escape latency to hit the target platform in MWM test in ICV-STZ rats on days 16 to 20. Swimming times of four trials per day for 5 days to each group are shown. Values are expressed as means ± S.E.M. (n=12). Average escape latency time (days 2-5) to find hidden platform was significantly prolonged in the ICV-STZ group compared with the control group (^#^p < 0.001 vs control group). AS605240 (5, 15 and 25 mg/kg) and donepezil treatment in ICV-STZ-induced groups dose-dependently and significantly reversed the ICV-STZ-induced learning deficit compared with STZ alone treated group (^**^p < 0.01 and ^***^p < 0.001, respectively). B: Percentage of time spent in the target quadrant in ICV-STZ rats. Values are expressed as means ± S.E.M. (n=12). The percentage of time spent in the target quadrant was significantly lesser in the ICV-STZ group compared with the control group (^#^p < 0.001). AS605240 (5, 15 and 25 mg/kg) and donepezil treatment in ICV-STZ-induced groups significantly attenuated STZ-induced memory deficits when compared with the STZ alone treated group (^*^P<0.05 L vs. control group; ^**^p < 0.01 and ^***^p < 0.001 respectively). STZ: Streptozotocin

The effect of AS605240 on escape latency in ICV-STZ-induced rats was evaluated by MWM task (Figure 3A(Fig. 3)). After 5 days of training, the escape latency to reach the submerged platform decreased in all groups. From day 2 to day 5, the average latency to locate the hidden platform in STZ group was significantly (p < 0.001) increased reaching maximum on fifth day of training trials when compared with the control group. This indicates a poorer learning and memory performance in STZ treated groups. Treatment with AS605240 at indicated doses (5, 15 and 25 mg/kg) significantly (p < 0.01 and p < 0.001) decreased escape latency compared with the STZ group. The standard donepezil treated group showed similar effects as compared with 25 mg/kg of AS605240 treated group (p < 0.001). Similar effects were observed in the spatial probe test with the platform removed. STZ treated rats had a significantly (p < 0.001) decreased amount of time spent in the target quadrant than the control group rats. However, the amount of time spent in the target quadrant by the rats in the AS605240 treated groups was much longer than that in the STZ group (Figure 3B(Fig. 3), p < 0.01 and p < 0.001), supporting a protective effect on learning and memory.

### Effect of AS605240 on LPO levels

**Figure 4:**
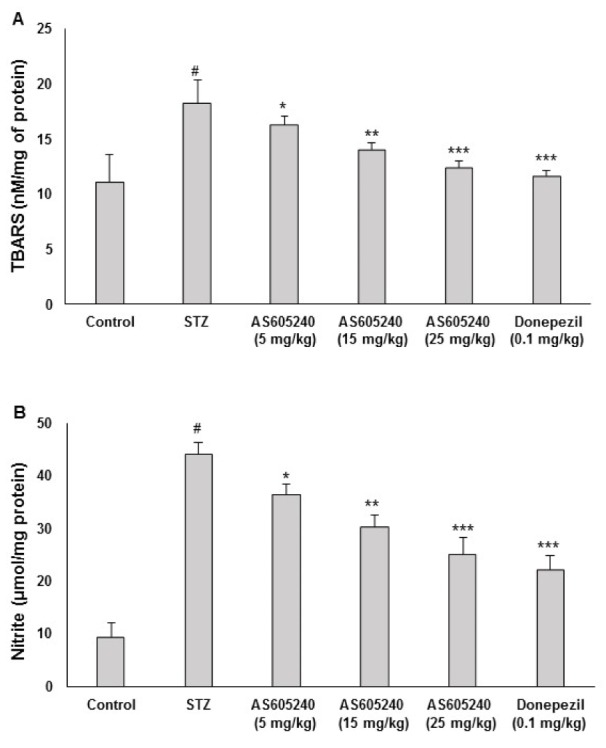
Effect of AS605240 on brain lipidperoxidation (LPO) and nitrite levels in ICV-STZ-induced rats. ICV-STZ treated rats showed a significant increase in brain TBARS (A) and nitrite (B) when compared to the control group. Administration of AS605240 (5,15 and 25 mg/kg) and donepezil (0.1 mg/kg; p.o.) prevented ICV-STZ induced increase in brain TBARS and nitrate levels dose-dependently and significantly when compared with STZ alone treated groups. The results are presented relative to control and the values are expressed as means ± S.E.M. (n=12). ^#^p < 0.001 when compared with control group and ^*^p < 0.05, ^**^p < 0.01 and ^***^p < 0,001, respectively when compared with STZ alone treated group. STZ: Streptozotocin, TBARS: Thiobarbituric acid reactive substances

The effect of AS605240 on brain LPO levels in ICV-STZ-induced rats was shown in Figure 4A(Fig. 4). ICV-STZ-administered rats had significantly increased level of TBARS in brain in comparison to control animals (p < 0.001). Administration of AS605240 (5, 15 and 25 mg/kg) prevented ICV-STZ induced increase in brain TBARS level significantly (p < 0.05, p< 0.01 and p < 0.001) in a dose-dependent fashion. The standard donepezil (0.1 mg/kg) treated group also decreased the TBARS level and was similar to AS605240 treated at 25 mg/kg treated group in the brains of ICV-STZ-administered rats (p < 0.001).

### Effect of AS605240 on nitrite levels

The effect of AS605240 on brain nitrite levels in ICV-STZ-induced rats was shown in Figure 4B(Fig. 4). ICV-STZ-administered rats significantly (p < 0.001) increased brain nitrite level in comparison to control animals. Administration of AS605240 (5,15 and 25 mg/kg) in ICV-STZ-induced animals significantly (p < 0.05, p< 0.01 and p < 0.001) reduced the brain nitrite level as compared to ICV-STZ-administered rats and the effects were dose-dependent. Similar results were observed in standard donepezil treated group and the effect was same as AS605240 treated at 25 mg/kg dose in ICV-STZ-treated rats (p < 0.001).

### Effect of AS605240 on GSH levels

**Figure 5:**
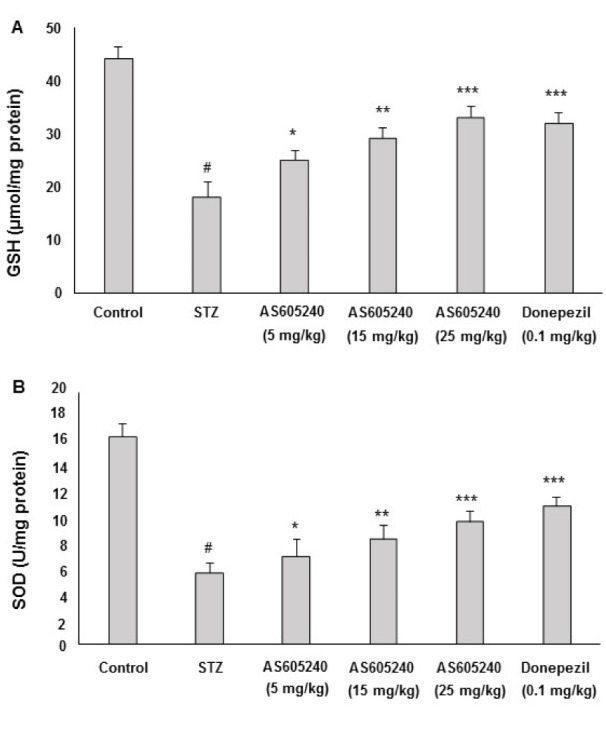
Effect of AS605240 on glutathione (GSH) and super oxide dismutase (SOD) in ICV-STZ-induced rats. ICV-STZ treated rats showed a significant decrease in GSH (A) and SOD (B) levels when compared to the control group. Administration of AS605240 (5, 15 and 25 mg/kg) and donepezil (0.1 mg/kg; p.o.) prevented ICV-STZ induced decrease in GSH and SOD levels significantly in a dose-dependent fashion when compared with STZ alone treated groups. The results are presented relative to control and the values are expressed as means ± S.E.M. (n=12). ^#^p < 0.001 when compared with control group and ^*^p < 0.05, ^**^p < 0.01 and ^***^p < 0,001, respectively when compared with STZ alone treated group. STZ: Streptozotocin. GSH: Glutathione, SOD: Superoxide dismutase

The effect of AS605240 on brain GSH levels in ICV-STZ-induced rats was shown in Figure 5A(Fig. 5). ICV-STZ administered rats showed significantly decreased level of GSH in the brain (p < 0.001). Treatment with AS605240 (5, 15 and 25 mg/kg) improved the depleted GSH level in brain as compared to STZ-treated rats significantly and concentration-dependently (p < 0.001). The standard donepezil (0.1 mg/kg) treated group also attenuated the altered levels of GSH and the effect was similar to AS605240 treated at 25 mg/kg dose (p < 0.001).

### Effect of AS605240 on SOD levels

The effect of AS605240 on SOD levels in ICV-STZ-induced rats was shown in Figure 5B(Fig. 5). ICV-STZ-administered rats showed significantly decreased activity of SOD in the brain in comparison to control animals (p < 0.001). However, administration of AS605240 (5, 15 and 25 mg/kg) significantly and dose-dependently attenuated the reduction in SOD activity as compared to ICV-STZ alone treated rats (p < 0.05, p < 0.01 and p < 0.001, respectively). The standard donepezil (0.1 mg/kg) treated group also attenuated the altered levels of SOD and the effect was similar to AS605240 treated at 25 mg/kg dose (p < 0.001).

### Effects of AS605240 on APP expression

**Figure 6:**
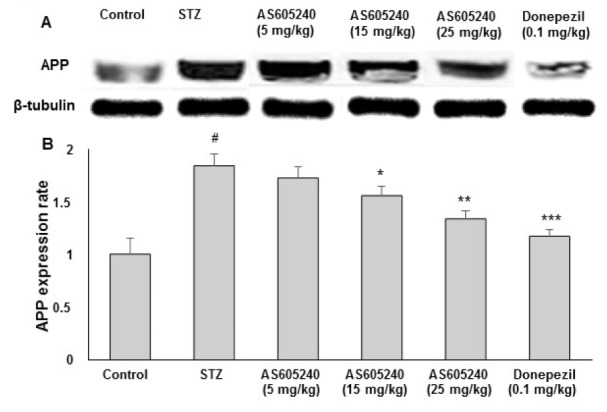
Effect of AS605240 on amyloid β precursor protein (APP) production in ICV-STZ-induced rat brain homogenates. APP protein levels in rat brain extracts were determined by immunoblot analysis (A) and quantified by image analysis (B). Equal loading of proteins was illustrated by β-tubulin bands. Values are expressed as means ± S.E.M. (n=12). ^#^p < 0.001 when compared with control group and ^*^p < 0.05, ^**^p < 0.01 and ^***^p < 0,001, respectively when compared with STZ alone treated group. APP: Amyloid β precursor protein, STZ: Streptozotocin

To determine whether AS605240 decreased Aβ levels by changing APP processing, we analyzed the expression of APP using WB analysis with anti-APP antibody (Figure 6A(Fig. 6)). The expression of APP was markedly increased in STZ-treated rats compared with the control group. However, in AS605240-treated rats (15 and 25 mg/kg), the APP expression was lowered dose-dependently (p < 0.05 at 15 mg/kg and p < 0.01 at 25 mg/kg) when compared with STZ-treated rats and similar effects were observed with the standard donepezil treatment (p < 0.01). Although the low dose of (5 mg/kg) of AS605240 treatment decreased the expression of APP when compared with STZ-induced group, the results were not statistically significant (Figure 6B(Fig. 6)). 

### Effect of AS605240 Immunohistostaining of Aß 

**Figure 7:**
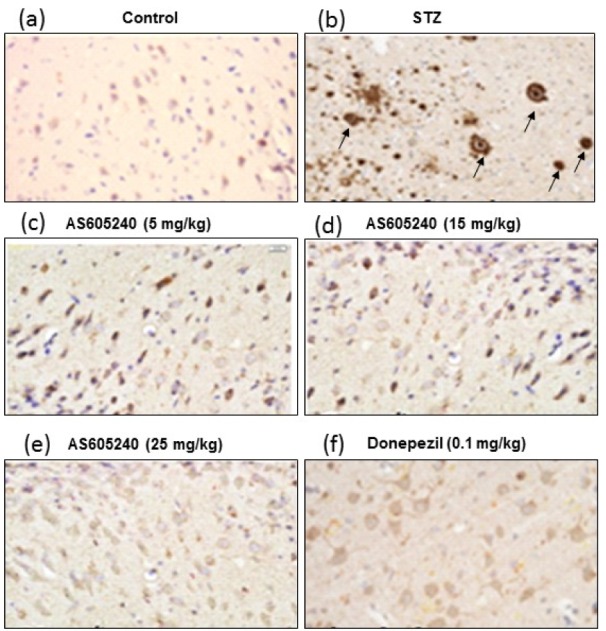
Effect of AS605240 on immunohistostaining in ICV-STZ-induced brain sections of rats. Immunoreactive signal of amyloid-β visualized by polyclonal rabbit antibody to β-amyloid 42. (a): Control group, (b): ICV-STZ-induced group, (c): AS605240 (5 mg/kg) treated group, (d): AS605240 (15 mg/kg) treated group, (e): AS605240 (25 mg/kg) treated group, and (f): Donepezil (0.1 mg/kg) treated group. The brown reaction product (DAB) shown in arrows could be seen markedly in STZ alone treated group (b) and weak staining in AS605240 and donepezil treated groups. Nissl counterstain. Scale bars 100 µm. Magnification 200X. STZ: Streptozotocin

To determine the accumulation of Aβ in ICV-STZ-induced rat brain tissue, immunohistostaining of Aβ was performed (Figure 7(Fig. 7)). Immunohistostaining using the polyclonal rabbit antibody to β-amyloid 42, which recognizes the Aβ peptide signal revealed that STZ injected rats had strong reaction with Aβ antibody with marked brown reaction products (DAB) in hippocampal areas compared with the control group which showed week reaction (Figure 7a-7b(Fig. 7)). In contrast, in AS605240 (5, 15 and 25 mg/kg) treated ICV-STZ-induced rats the Aβ peptide signal was decreased with increasing doses (Figure 7c-7e(Fig. 7)). These results suggested that the AS605240 may promote clearance of soluble intracellular Aβ deposits. The standard donepezil (0.1 mg/kg) treated group also decreased the Aβ signal and the effect was similar to AS605240 treated at 25 mg/kg dose (Figure 7f(Fig. 7)).

## Discussion

The present study was focused on the protective effects of AS605240, a potential PI3K inhibitor on cognitive deficits, antioxidative defense parameters and APP expression in ICV-STZ induced sporadic AD model in rats. It is well documented that a single dose of ICV-STZ (3 mg/kg) induced rats showed significant impairments in memory and learning in behavioral test and further altered the antioxidative defense markers and increased the protein expression of APP (Fanoudi et al., 2018[17]). In agreement with the published data, in the present study ICV-STZ induced pathological alterations simulated sporadic AD-like pathology by altering memory and learning, oxidative stress and as well as increased expression of APP. However, treatment with AS605240 (5, 15 and 25 mg/kg) significantly and concentration-dependently ameliorated the cognitive impairments induced by ICV-STZ and further ameliorated the ICV-STZ-induced alterations in biochemical parameters including antioxidative defense parameters and the protein expression of APP.

Several studies on AS605240 suggested a range of concentrations varying from as low as 5 mg/kg to as high as 1000 mg/kg for 40-60 days is safe and non-toxic in experimental animals (Salkovic-Petrisic et al., 2011[47]; Passos et al., 2010[39]). Oral administration of AS605240 (5-50 mg/kg) has been shown to be pharmacologically effective in several animal disease models, and is able to cross the blood brain barrier (BBB) in the normal brain and damaged BBB in the ischemic brain (Jin et al., 2019[27]). Further, reports also revealed that AS605240 (10 mg/kg) exhibited anti-inflammatory effects by significantly reducing brain water content within peri-resection brain tissues and improved the neurological performance in surgical brain injury animal models (Huang et al., 2015[23]). Based on these reports, in the present study the concentrations of 5, 15 and 25 mg/kg body weight of AS605240 were employed. Notably, no obvious side effects or toxic reactions were observed in the groups treated with indicated concentrations of AS605240.

In behavioral studies, ICV-STZ induced group showed impairment in learning and memory by reduction in latency time to enter the dark side of the chamber in passive avoidance task. Further, in MWM behavior task, ICV-STZ-induced group showed an increase in the escape latency time and reduction in the percentage of time spent in the target quadrant. The results are consistent with previous reported studies indicating cognitive deficits in ICV-STZ-induced rats (Javed et al., 2012[25]; Mansouri et al., 2013[31]; Chen et al., 2016[8]). However, treatment with AS605240 reversed these changes in both the behavioral tasks tested suggesting improved learning and memory abilities.

Depletion in oxidative defense mechanisms *via* free radical attack initiates a cascade of debilitating events that progress brain aging and AD symptoms (Christen, 2000[10]; Liu et al., 2003[30]; Barnham et al., 2004[3]). LPO, and imbalance in the antioxidative defense markers including the GSH, SOD and nitrite in the brain increases the free radical mediated progression of AD pathogenesis and memory impairments (Sultana et al., 2013[55]; Saharan and Mandal, 2014[46]; Wink et al., 2001[60]; Javed et al., 2012[25]; Susswein et al., 2004[56]). Earlier reports indicated that ICV-STZ infusion enhanced the LPO and nitrite levels and decreased the antioxidant marker status of GSH and SOD in experimental animals (Ishrat et al., 2009[24]; Mehla et al., 2012[33]). In agreement with the previous reports, in the present study, the altered levels of LPO, GSH, SOD and nitrite levels were attenuated in brain tissues demonstrating antioxidant defense mechanism of AS605240.

It was well documented that Aβ accumulation as a key role in the mechanism of neuron damage and cognitive dysfunction and formation of amyloid plaques is considered as one of the main hallmarks of AD (Harrington et al., 2015[22]). Earlier studies indicated that ICV-STZ induced rats showed a decrease in brain weight, cognitive decline, a significant increase in hippocampal Aβ with increased expression of APP (Correia et al., 2013[12]; Lindberg et al., 2012[29]). Therefore, in the present study, the expression of APP was evaluated using WB and the deposition of Aβ was visualized immunohistochemically. As expected, WB analysis in ICV-STZ injected rat brain tissues clearly increased the expression of APP and treatment with AS605240 showed a reduction of APP expression. Further, immunoreactive signal of Aβ showed marked appearance of the brown reaction product (DAB) in ICV-STZ-induced group and AS605240 treated groups (5, 15 and 25 mg/kg) showed dose-dependent decrease in the reaction products with weak Aβ signal with increased concentrations of AS605240. 

In the present study, donepezil was used as a standard as it has been well reported to rescue learning and memory related behavioral impairments, attenuate the altered antioxidative defense parameters and reduce Aβ production (Sonkusare et al., 2005[52]; Nordberg, 2006[37]; Saxena et al., 2008[48]). Consistent with the previous reports, the protective effects of AS605240 treated at 25 mg/kg were comparable to those of donepezil on learning and memory impairments, as well as antioxidant capacities. Earlier reports indicated that AS605240 useful in general cognitive enhancement on the basis of its ability to improve learning and memory in mice induced by β-amyloid 1-40 peptide (Passos et al., 2010[39]). AS605240 has also been suggested to improve the neurological function score, reduce the infarct size and decrease astrocyte activation in the stroke-related injury in the mouse model of transient intraluminal middle cerebral artery occlusion thereby aiding in the repair and remodeling of neurons (Shang et al., 2019[50]). Further, AS605240 was reported to attenuate tissue-type plasminogen activator-induced brain hemorrhage and improved microvascular patency after embolic stroke in rats likely contributing to the neuroprotective effect of AS605240 (Jin et al., 2019[27]). Based on the above literature and the present data, our study supports the notion that the PI3K inhibitor, AS605240 can be considered as a neuroprotective therapeutic target in the treatment of AD. However, the detailed neuroprotective mechanism that mediates the interplay between the inhibition of PI3Kγ and the protective outcome of AS605240 against ICV-STZ-induced sporadic AD needs further investigation.

In conclusion, our study demonstrated that AS605240 has protective effect against ICV-STZ-induced behavioral and biochemical parameters in rats. AS605240 administration to ICV-STZ induced sporadic AD rat improves memory by its potential PI3K inhibition and antioxidative defense mechanisms proving its therapeutic potential in the treatment and management of sporadic AD. 

## Notes

Ramesh Alluri and Sushruta Koppula (College of Biomedical and Health Science, Konkuk University, Chungju-Si, Chungbuk Do, 27478, Republic of Korea; Tel: +824384403609, E-mail: koppula@kku.ac.kr) equally contributed as corresponding authors.

## Acknowledgements

This work was supported by the grant from the Department of Science and Technology (DST), Ministry of Science and Technology, Government of India, New Delhi and partly by Konkuk University, South Korea. 

## Conflict of interest

The authors declare that they have no conflict of interest. 

## References

[1] Azzi J, Moore RF, Elyaman W, Mounayar M, El Haddad N, Yang S (2012). The novel therapeutic effect of phosphoinositide 3-kinase-γ inhibitor AS605240 in autoimmune diabetes. Diabetes.

[2] Barber DF, Bartolomé A, Hernandez C, Flores JM, Redondo C, Fernandez-Arias C (2005). PI3Kgamma inhibition blocks glomerulonephritis and extends lifespan in a mouse model of systemic lupus. Nat Med.

[3] Barnham KJ, Masters CL, Bush AI (2004). Neurodegenerative diseases and oxidative stress. Nat Rev Drug Discov.

[4] Beauchamp C, Fridovich I (1971). Superoxide dismutase: improved assays and an assay applicable to acrylamide gels. Anal Biochem.

[5] Brookmeyer R, Gray S, Kawas C (1998). Projections of Alzheimer’s disease in the United States and the public health impact of delaying disease onset. Am J Public Health.

[6] Camps M, Rückle T, Ji H, Ardissone V, Rintelen F, Shaw J (2005). Blockade of PI3Kgamma suppresses joint inflammation and damage in mouse models of rheumatoid arthritis. Nat Med.

[7] Casey DA, Antimisiaris D, O’Brien J (2010). Drugs for Alzheimer’s disease: are they effective?. P & T.

[8] Chen L, Zhang Y, Li D, Zhang N, Liu R, Han B (2016). Everolimus (RAD001) ameliorates vascular cognitive impairment by regulating microglial function via the mTORC1 signaling pathway. J Neuroimmunol.

[9] Chen YLZ, Blanchard J, Dai CL, Sun S, Lee MH, Grundke-Iqbal I (2013). A non-transgenic mouse model (icv-STZ Mouse) of Alzheimer’s disease: similarities to and differences from the transgenic model (3xTg-AD Mouse). Mol Neurobiol.

[10] Christen Y (2000). Oxidative stress and Alzheimer disease. Am J Clin Nutr.

[11] Correia SC, Santos RX, Perry G, Zhu X, Moreira PI, Smith MA (2011). Insulin-resistant brain state: the culprit in sporadic Alzheimer’s Disease. Ageing Res Rev.

[12] Correia SC, Santos RX, Santos MS, Casadesus G, Lamanna JC, Perry G (2013). Mitochondrial abnormalities in a streptozotocin-induced rat model of sporadic Alzheimer's disease. Curr Alzheimer Res.

[13] Cushing TD, Metz DP, Whittington DA, McGee LR (2012). PI3Kδ and PI3Kγ as targets for autoimmune and inflammatory diseases. J Med Chem.

[14] DeKosk ST (2003). Pathology and pathways of Alzheimer’s disease with an update on new developments in treatment. J Am Geriatr Soc.

[15] Dhull DK, Jindal A, Dhull RK, Aggarwal S, Bhateja D, Padi SS (2012). Neuroprotective effect of cyclooxygenase inhibitors in ICV-STZ induced sporadic Alzheimer's disease in rats. J Mol Neurosci.

[16] Ellman GL (1959). Tissue sulfhydryl groups. Arch Biochem Biophys.

[17] Fanoudi S, Hosseini M, Alavi MS, Boroushaki MT, Hosseini A, Sadeghnia HR (2018). Everolimus, a mammalian target of rapamycin inhibitor, ameliorated streptozotocin-induced learning and memory deficits via neurochemical alterations in male rats. EXCLI J.

[18] Giacobini E, Giacobini E, Pepeu G (2006). Cholinesterases in human brain: the effect of cholinesterase inhibitors on Alzheimer’s disease and related disorders. The brain cholinergic system in health and disease.

[19] Green LC, Wagner DA, Glogowski J, Skipper PL, Wishnok JS, Tannenbaum SR (1982). Analysis of nitrate, nitrite and (15N) nitrate in biological fluids. Anal Biochem.

[20] Griffin RJ, Moloney A, Kelliher M, Johnston JA, Ravid R, Dockery P (2005). Activation of Akt/PKB, increased phosphorylation of Akt substrates and loss and altered distribution of Akt and PTEN are features of Alzheimer's disease pathology. J Neurochem.

[21] Hardy J, Selkoe DJ (2002). The amyloid hypothesis of Alzheimer’s disease: progress and problems on the road to therapeutics. Science.

[22] Harrington KD, Lim YY, Gould E, Maruff P (2015). Amyloid‑beta and depression in healthy older adults: A systematic review. Aust N Z J Psychiatry.

[23] Huang L, Sherchan P, Wang Y, Reis C, Applegate RL, Tang J (2015). Phosphoinositide 3-kinase gamma contributes to neuroinflammation in a rat model of surgical brain injury. J Neurosci.

[24] Ishrat T, Hoda MN, Khan MB, Yousuf S, Ahmad M, Khan MM (2009). Amelioration of cognitive deficits and neurodegeneration by curcumin in rat model of sporadic dementia of Alzheimer’s type (SDAT). Eur Neuropsychopharmacol.

[25] Javed H, Khan M, Ahmad A, Vaibhav K, Ahmad M, Khan A (2012). Rutin prevents cognitive impairments by ameliorating oxidative stress and neuroinflammation in rat model of sporadic dementia of Alzheimer type. Neuroscience.

[26] Jin K, Song LF, He CM, Wang ZL, Hu XH, Wu XH (2009). Intervention effect of PI3Kgamma inhibitor AS605240 on autoimmune myocarditis in mice. Sichuan Da Xue Xue Bao Yi Xue Ban.

[27] Jin R, Xiao AY, Li J, Wang M, Li G (2019). PI3Kγ (Phosphoinositide 3-Kinase-γ) inhibition attenuates tissue-type plasminogen activator-induced brain hemorrhage and improves microvascular patency after embolic stroke. Hypertension.

[28] Kraska ASM, Dorieux O, Joseph-Mathurin N, Bourrin E, Petit F, Jan C (2012). In vivo cross-sectional characterization of cerebral alterations induced by intracerebroventricular administration of streptozotocin. PLoS One.

[29] Lindberg O, Walterfang M, Looi JC, Malykhin N, Ostberg P, Zandbelt B (2012). Hippocampal shape analysis in Alzheimer’s disease and frontotemporal lobar degeneration subtypes. J Alzheimers Dis.

[30] Liu R, Liu IY, Bi X, Thompson RF, Doctrow SR, Malfroy B (2003). Reversal of age-related learning deficits and brain oxidative stress in mice with superoxide dismutase/catalase mimetics. Proc Natl Acad Sci USA.

[31] Mansouri MT, Naghizadeh B, Ghorbanzadeh B, Farbood Y, Sarkaki A, Bavarsad K (2013). Gallic acid prevents memory deficits and oxidative stress induced by intracerebroventricular injection of streptozotocin in rats. Pharmacol Biochem Behav.

[32] Masliah E, Mallory M, Hansen L, DeTeresa R, Alford M, Terry R (1994). Synaptic and neuritic alterations during the progression of Alzheimer’s disease. Neurosci Lett.

[33] Mehla J, Pahuja M, Dethe SM, Agarwal A, Gupta YK (2012). Amelioration of intracerebroventricular streptozotocin induced cognitive impairment by Evolvulus alsinoides in rats: in vitro and in vivo evidence. Neurochem Int.

[34] Mehla J, Pahuja M, Gupta YK (2013). Streptozotocin-induced sporadic Alzheimer's disease: selection of appropriate dose. J Alzheimers Dis.

[35] Morris, R (1984). Developments of a water-maze procedure for studying spatial learning in the rat. J. Neurosci Methods.

[36] Nitsch R, Hoyer S (1991). Local action of the diabetogenic drug, streptozotocin, on glucose and energy metabolism in rat brain cortex. Neurosci Lett.

[37] Nordberg A (2006). Mechanisms behind the neuroprotective actions of cholinesterase inhibitors in Alzheimer disease. Alzheimer Dis Assoc Disord.

[38] Ohkawa H, Ohishi N, Yagi K (1979). Assay for lipid peroxidation in animal tissues by thiobarbituric acid reaction. Anal Biochem.

[39] Passos GF, Figueiredo CP, Prediger RD, Silva KA, Siqueira JM, Duarte FS (2010). Involvement of phosphoinositide 3-kinase gamma in the neuro-inflammatory response and cognitive impairments induced by beta-amyloid 1-40 peptide in mice. Brain Behav Immun.

[40] Paxinos G, Watson C (1986). The rat brain in stereotaxic coordinate.

[41] Peng XD, Wu XH, Chen LJ, Wang ZL, Hu XH, Song LF (2010). Inhibition of phosphoinositide 3-kinase ameliorates dextran sodium sulfate-induced colitis in mice. J Pharmacol Exp Ther.

[42] Ponce-Lopez T, Liy-Salmeron G, Hong E, Meneses A (2011). Lithium, phenserine, memantine and pioglitazone reverse memory deficit and restore phospho-GSK3beta decreased in hippocampus in intracerebroventricular streptozotocin induced memory deficit model. Brain Res.

[43] Prickaerts J, Fahrig T, Blokland A (1999). Cognitive performance and biochemical markers in septum, hippocampus and striatum of rats after an i.c.v. injection of streptozotocin: a correlation analysis. Behav Brain Res.

[44] Raghavendra V, Kulkarni SK (2001). Possible antioxidant mechanism in melatonin reversal of aging and chronic ethanol-induced amnesia in plus-maze and passive avoidance memory tasks. Free Radic Biol. Med.

[45] Ramezani M, Darbandi N, Khodagholi F, Hashemi A (2016). Myricetin protects hippocampal CA3 pyramidal neurons and improves learning and memory impairments in rats with Alzheimer's disease. Neural Regen Res.

[46] Saharan S, Mandal PK (2014). The emerging role of glutathione in Alzheimer’s disease. J Alzheimers Dis.

[47] Salkovic-Petrisic M, Osmanovic-Barilar J, Brückner M, Hoyer S, Arendt T, Riederer P (2011). Cerebral amyloid angiopathy in streptozotocin rat model of sporadic Alzheimer’s disease: a long-term follow up study. J Neural Transm.

[48] Saxena G, Singh SP, Agrawal R, Nath C (2008). Effect of donepezil and tacrine on oxidative stress in intracerebral streptozotocin-induced model of dementia in mice. Eur J Pharmacol.

[49] Selkoe DJ (2001). Alzheimer’s disease: genes, proteins, and therapy. Physiol Rev.

[50] Shang S, Liu L, Wu X, Fan F, Hu E, Wang L (2019). Inhibition of PI3Kγ by AS605240 protects tMCAO mice by attenuating pro-inflammatory signaling and cytokine release in reactive astrocytes. Neuroscience.

[51] Song LF, Jiang W, Qing Y, Hu XH, Li Y, Tong QY (2011). The antagonistic effect of PI3K-gamma inhibitor AS605240 on cardiac hypertrophy and cardiac fibrosis induced by isoproterenol in rats. Sichuan Da Xue Xue Bao Yi Xue Ban.

[52] Sonkusare S, Srinivasan K, Kaul C, Ramarao P (2005). Effect of donepezil and lercanidipine on memory impairment induced by intracerebroventricular streptozotocin in rats. Life Sci.

[53] Spitzenberg V, König C, Ulm S, Marone R, Röpke L, Müller JP (2010). Targeting PI3K in neuroblastoma. J Cancer Res Clin Oncol.

[54] Stuchbury G, Münch G (2005). Alzheimer’s associated inflammation, potential drug targets and future therapies. J Neural Transm.

[55] Sultana R, Perluigi M, Butterfield DA (2013). Lipid peroxidation triggers neurodegeneration: a redox proteomics view into the Alzheimer disease brain. Free Radic Biol Med.

[56] Susswein AJ, Katzoff A, Miller N, Hurwitz I (2004). Nitric oxide and memory. Neuroscientist.

[57] Tanzi RE, Bertram L (2005). Twenty years of the Alzheimer’s disease amyloid hypothesis: a genetic perspective. Cell.

[58] Wang ZL, Wu XH, Song LF, Wang YS, Hu XH, Luo YF (2009). Phosphoinositide 3-kinase gamma inhibitor ameliorates concanavalin A-induced hepatic injury in mice. Biochem Biophys Res Commun.

[59] Wei X, Han J, Chen ZZ, Qi BW, Wang GC, Ma YH (2010). A phosphoinositide 3-kinase-gamma inhibitor, AS605240 prevents bleomycin-induced pulmonary fibrosis in rats. Biochem Biophys Res Commun.

[60] Wink DA, Miranda KM, Espey MG, Pluta RM, Hewett SJ, Colton C (2001). Mechanisms of the antioxidant effects of nitric oxide. Antioxid Redox Signal.

[61] Zhang C, Browne A, Divito JR, Stevenson JA, Romano D, Dong Y (2010). Amyloid-β production via cleavage of amyloid-β protein precursor is modulated by cell density. J Alzheimers Dis.

[62] Zhao WQ, De Felice FG, Fernandez S, Chen H, Lambert MP, Quon MJ (2008). Amyloid beta oligomers induce impairment of neuronal insulin receptors. FASEB J.

